# Personal and trip characteristics associated with safety equipment use by injured adult bicyclists: a cross-sectional study

**DOI:** 10.1186/1471-2458-12-765

**Published:** 2012-09-11

**Authors:** Kay Teschke, Jeff R Brubacher, Steven M Friedman, Peter A Cripton, M Anne Harris, Conor CO Reynolds, Hui Shen, Melody Monro, Garth Hunte, Mary Chipman, Michael D Cusimano, Nancy Smith Lea, Shelina Babul, Meghan Winters

**Affiliations:** 1 School of Population and Public Health, 2206 East Mall, University of British Columbia, Vancouver, BC, Canada; 2 Department of Emergency Medicine, University of British Columbia, Vancouver, BC, Canada; 3 Emergency Medicine, University Health Network, Toronto, ON, Canada; 4 Department of Mechanical Engineering, ICORD and the Brain Research Centre, University of British Columbia, Vancouver, BC, Canada; 5 Occupational Cancer Research Centre, Toronto, ON, Canada; 6 Liu Institute for Global Issues, University of British Columbia, Vancouver, BC, Canada; 7 School of Public Health, University of Toronto, Toronto, ON, Canada; 8 Toronto Centre for Active Transportation, Toronto, ON, Canada; 9 British Columbia Injury Research and Prevention Unit, Vancouver, BC, Canada; 10 Faculty of Health Sciences, Simon Fraser University, Burnaby, BC, Canada

**Keywords:** Active transport, Bicycle safety, Visibility, Bicycle helmet

## Abstract

**Background:**

The aim of this study was to estimate use of helmets, lights, and visible clothing among cyclists and to examine trip and personal characteristics associated with their use.

**Methods:**

Using data from a study of transportation infrastructure and injuries to 690 adult cyclists in Toronto and Vancouver, Canada, we examined the proportion who used bike lights, conspicuous clothing on the torso, and helmets on their injury trip. Multiple logistic regression was used to examine associations between personal and trip characteristics and each type of safety equipment.

**Results:**

Bike lights were the least frequently used (20% of all trips) although they were used on 77% of trips at night. Conspicuous clothing (white, yellow, orange, red) was worn on 33% of trips. Helmets were used on 69% of trips, 76% in Vancouver where adult helmet use is required by law and 59% in Toronto where it is not. Factors positively associated with bike light use included night, dawn and dusk trips, poor weather conditions, weekday trips, male sex, and helmet use. Factors positively associated with conspicuous clothing use included good weather conditions, older age, and more frequent cycling. Factors positively associated with helmet use included bike light use, longer trip distances, hybrid bike type, not using alcohol in the 6 hours prior to the trip, female sex, older age, higher income, and higher education.

**Conclusions:**

In two of Canada’s largest cities, helmets were the most widely used safety equipment. Measures to increase use of visibility aids on both daytime and night-time cycling trips may help prevent crashes.

## Background

Bicycling injuries are a concern both because of the direct harm they cause individuals and because concerns about safety are a deterrent to use of this healthy mode of transportation, especially in North America [1-3]. In the United States, collisions with motor vehicles result in about 700 fatalities and 48,000 police-reported injuries per year among cyclists [4]. In Canada, with a population about one-tenth of the US, collisions with motor vehicles result in about 50 fatalities and 450 serious injuries (requiring hospitalization) per year among cyclists [5]. These data do not account for all injuries to cyclists, since they do not tally crashes not involving motor vehicles, and they may also miss some that do [6].

As outlined in William Haddon’s original work on traffic injury epidemiology, there are many potential approaches to injury reduction. Measures can be directed at the individual cyclist or the cycling environment, and can be focussed on pre-event prevention or post-event mitigation [7]. A number of authors have noted a difference in emphasis in bicycling safety between northern Europe and North America, with an environment focus dominant in the former and an individual focus dominant in the latter [8,9]. Within individual-based safety measures, there is a range of possible options, including those aimed at crash prevention (e.g., lights) and those focussed on injury mitigation (e.g., helmets). A number of studies have measured use of individual-based safety equipment by cyclists [10-25], though few have documented use of multiple types of equipment in the same population. Fewer still have examined characteristics (e.g., weather conditions, cyclist age) associated with use of safety equipment [10-12,14-18,20,22,25]. Better understanding could help inform priorities for improvement, interventions to improve uptake, and the relative safety potential of these individual-based measures vis-à-vis population-based alternatives such as bicycle-dedicated infrastructure.

As part of a study of 690 cyclists injured in two of Canada’s largest cities, Toronto and Vancouver, we collected data on use of lights, high conspicuity clothing on the torso, and helmets. In addition, we collected data on trip and personal characteristics that allowed us to examine factors associated with use of these types of safety equipment.

## Methods

The study methods were reviewed and approved by the human subjects ethics review boards of the University of British Columbia, the University of Toronto, St. Paul’s Hospital, Vancouver General Hospital, St. Michael’s Hospital, and the University Health Network (Toronto General Hospital and Toronto Western Hospital). All participants gave informed consent before taking part in the study.

Methods of study conduct have been described in detail elsewhere [26]. The study population consisted of adult (≥ 19 years) residents of Toronto and Vancouver who were injured while riding a bicycle in the city and treated within 24 hours in the emergency departments of the hospitals listed above between May 18, 2008 and November 30, 2009.

Eligible participants were interviewed in person by trained interviewers, using a structured questionnaire (http://cyclingincities.spph.ubc.ca/files/2011/10/InterviewFormFinal.pdf) as soon as possible after the injury to maximize recall. Questions related to safety equipment use were the following (asked in this order):

Did you have a back light that was turned on during this trip?

Did you have a front light that was turned on during this trip?

What colour was the clothing on your upper body?

What colour was the helmet you were wearing?

Questions about front and back lights were combined and if at least one light was turned on, assigned a “yes”. The following torso clothing colours were classified as highly visible based on evidence of conspicuity from the study of Hagel *et al.*[18]: white, yellow, orange, and red. Those who reported a helmet colour were classified as wearing a helmet. “Don’t know” or “refused” responses for all questions were grouped with the “no” category, to provide a conservative estimate of the prevalences of safety equipment use. Only highly visible clothing on the torso had large numbers of “don’t know” responses; we repeated analyses (removing “don’t know” responses) to determine whether this conservative classification had an impact on the results.

The interview also collected data on trip characteristics (weather conditions, time of day, day of week, season, trip distance, trip purpose, bike type used, alcohol use in the 6 hours prior to the trip, drug use in the 6 hours prior, sleep in the 24 hours prior, and whether the participant was cycling with a companion) and personal characteristics (age, sex, education, income, employment, cycling frequency, and whether the participant had a driver’s license, was an experienced cyclist, had taken a cycling training course, and had children in the household).

Unconditional multiple logistic regression was used to examine associations between the use of each type of safety equipment (bike lights, highly visible clothing on the torso, or helmet; as dependent variables) and the following independent variables: city; all trip and personal characteristics listed above; and the two types of safety equipment that were not the dependent variable in the analysis. We used backwards selection to construct multiple logistic regression models, starting by offering all variables of interest. Based on results of the Wald test for each variable, the variable with the highest p-value was removed and the model refit with the remaining variables until all variables in the model were statistically significant at the p < 0.05 level. Data analyses were performed using SAS 9.2 (SAS Institute, Cary, NC). In this paper we present the unadjusted and adjusted results for the variables in the final models. The results for full models with all variables included (prior to backwards selection) are available from the authors.

## Results

Details on the recruitment process are available elsewhere [26]. In brief, 2,335 injured cyclists attended one of the five study emergency departments during the study period. Of these, 927 were deemed ineligible, 741 deemed eligible and 690 participated (414 in Vancouver, 276 in Toronto). There were 667 with unknown eligibility (543 not contacted, 124 refusals). Participants represented 93.1% of those confirmed to be eligible and 66.5% of those estimated to be eligible (based on the proportion eligible among those contacted). The most common reasons for ineligibility were not being a resident of the study city and being injured outside the city.

Table 1 lists selected participant and trip characteristics. Most participants were men, younger than 40, well educated, employed, earned more than $50,000 a year, were regular cyclists, and had a driver’s license. Most of the injury trips were short and utilitarian in nature. Few participants had taken alcohol or drugs in the 6 hours prior to the trip, were sleep deprived, or were travelling with a companion. Most of the injury events were collisions (i.e., involved hitting a vehicle, object, surface, person or animal) rather than falls, and almost half involved a motor vehicle (one-third directly and 14% indirectly in avoidance manoeuvres). Most occurred at non-intersection locations.

**Table 1:** Characteristics of the study participants and the bicycling trips when they were injured (N = 690)

**Characteristic**	**Number (%)**
Male	410 (59.4%)
Female	280 (40.6%)
Age (of N = 685 reporting)	
19 to 29 years	250 (36.5%)
30 to 39 years	177 (25.8%)
40 to 49 years	108 (15.8%)
50 to 59 years	91 (13.3%)
60 to 69 years	49 (7.2%)
≥ 70 years	10 (1.5%)
Completed post-secondary diploma or degree	518 (75.1%)
Employed	546 (79.1%)
Income greater than $50,000 (of N = 610 reporting)	341 (55.9%)
Had children in their household	104 (15.1%)
Regular cyclist (cycled ≥ 52 times per year)	608 (88.1%)
Considered themselves an experienced cyclist	529 (76.7%)
Had taken an urban cycling training course	42 (6.1%)
Had bike maintained in the last 6 months	525 (76.1%)
Had a driver’s license	620 (89.9%)
Trip < 5 km	470 (68.1%)
Trip purpose	
Commute to or from work or school	287 (41.6%)
For exercise or recreation	177 (25.7%)
For social reasons (e.g., movies, visit friends)	159 (23.0%)
For personal business (e.g., shopping, doctor’s visit)	126 (18.3%)
During work	17 (2.5%)
Alcohol or drug use in 6 hours prior to trip	
Alcohol	73 (10.5%)
Medications	52 (7.5%)
Recreational drugs	25 (3.6%)
Had less than 6 hours of sleep in 24 hours prior to trip	23 (3.3%)
Cycling with a companion	109 (15.8%)
Injury circumstances	
Collision	497 (72.0%)
Fall	193 (28.0%)
Motor vehicle involved	331 (48.0%)
Crash at an intersection	211 (30.6%)

Use of safety equipment is outlined in Table 2. Lighting was the least frequently used, with 135 (19.6%) participants indicating they had at least one light turned on, including 96 using both lights, 25 with only the back light on and 14 with only the front light on. Seven participants responded “don’t know”. Responses about clothing indicated that 230 (33.3%) wore white, yellow, orange or red on their torso. There were 56 participants who responded “don’t know” and three who were not wearing clothing on the torso. We did not directly ask for information about use of reflective material, which is visible at night under illumination. It was self-reported by 59 individuals, 34 of whom were wearing colours that were not classified as conspicuous. Because this information was not directly solicited in questioning, reflective material use is likely to have been under-reported. Helmets were the most frequently used safety equipment with 478 (69.3%) participants indicating their helmet colour. Two responded “don’t know” and one person refused to answer.

**Table 2:** Safety equipment use on the trip: bike lights; visible clothing; and helmets

	**At least one bike light turned on**	**Highly visible clothing worn on the torso**	**Helmet worn**
	Yes/No*	**Yes/No**	**Yes/No**
**At least one bike light turned on**			
Yes	135/0	49/86	102/33
No	0/555	181/374	376/179
**Highly visible clothing worn on the torso**			
Yes	49/181	230/0	169/61
No	86/374	0/460	309/151
**Helmet worn**			
Yes	102/376	169/309	478/0
No	33/179	61/151	0/212

* Note that all “no” categories also include those who didn’t know or refused to answer the question;

· for “at least one bike light turned on”, 7 participants (1.01%) indicated     they didn’t know;

· for “highly visible clothing worn on the torso”, 56 indicated they didn’t     know (8.1%), and;

· for “helmet worn”, 2 indicated they didn’t know and 1 refused (0.44%);

Table 3 shows the logistic regression results for factors associated with having at least one bike light turned on. The strongest relationships were for time of day: only 6% of participants had lights on during the daytime, *versus* 44% at dawn or dusk, and 77% at night. Dull weather also prompted light use, with strong associations for cloud cover, fog, mist, rain or snow. Those on weekend trips and women were less likely to use lights (even after adjusting for weather and time of day). Helmet use was positively associated with use of lights.

**Table 3:** Associations between whether at least one bike light was turned on and trip or personal characteristics, variables retained in adjusted analysis only, each variable on its own (unadjusted) and in multiple logistic regression (adjusted)

	**At least one bike light turned on**			
	**Yes/No**	(% Yes/% No)*	**Unadjusted Odds Ratio (95% Confidence Interval)**	**Adjusted Odds Ratio (95% Confidence Interval)**
**Trip time of day**				
Day	32/503	(6/94)	1 (reference)	1 (reference)
Dawn or dusk	22/28	(44/56)	**12.6 (6.49 – 24.6)**	**13.2 (6.42 – 27.2)**
Night	81/24	(77/23)	**50.2 (30.0 – 90.2)**	**71.1 (35.8 – 141)**
**Trip weather type**				
Clear sky	63/414	(13/87)	1 (reference)	1 (reference)
Cloud cover	39/93	(30/70)	**2.91 (1.83 – 4.61)**	**3.43 (1.83 – 6.41)**
Fog, mist, rain or snow	25/35	(42/58)	**4.85 (2.72 – 8.65)**	**3.07 (1.33 – 7.10)**
Wind	3/11	(21/79)	2.03 (0.55 – 7.61)	1.75 (0.30 – 10.4)
**Trip day of week**				
Weekday	109/422	(21/79)	1 (reference)	1 (reference)
Weekend	26/133	(16/84)	0.73 (0.45 – 1.19)	**0.48 (0.24 – 0.97)**
**Sex**				
Male	92/318	(22/78)	1 (reference)	1 (reference)
Female	43/237	(15/85)	**0.60 (0.40 – 0.91)**	**0.56 (0.32 – 0.99)**
**Helmet worn during trip**				
No	33/179	(16/84)	1 (reference)	1 (reference)
Yes	102/376	(21/79)	1.45 (0.93 – 2.25)	**3.15 (1.61 – 6.16)**

Significant associations in bold.

* Row percent.

Few variables showed associations with wearing highly visible clothing on the torso (Table 4). Cold, wet weather was associated with lower odds of wearing conspicuous clothing. Older adults (50 to 59 years) and those who were more frequent cyclists were more likely to wear such clothing. In a repeat of the analyses for highly visible clothing, excluding the 56 participants who could not recall the colour of the clothing they wore, the variables associated and their odds ratios and confidence intervals were nearly identical to the adjusted analyses reported in Table 4.

**Table 4:** Associations between whether highly visible clothing was worn on the torso and trip or personal characteristics, variables retained in adjusted analysis only, each variable on its own (unadjusted) and in multiple logistic regression (adjusted)

	**Highly visible clothing worn on the torso**			
	**Yes/No**	(% Yes/% No)*	**Unadjusted Odds Ratio (95% Confidence Interval)**	**Adjusted Odds Ratio (95% Confidence Interval)**
**Trip weather type**				
Clear sky	172/305	(36/64)	1 (reference)	1 (reference)
Cloud cover	44/88	(33/67)	0.88 (0.53 – 1.33)	0.85 (0.56 – 1.29)
Fog, mist, rain or snow	10/50	(16/84)	**0.36 (0.18 – 0.72)**	**0.33 (0.16 – 0.68)**
Wind	4/10	(29/71)	0.79 (0.24 – 2.60)	0.85 (0.26 – 2.86)
**Age**				
19 - 29	78/185	(30/70)	1 (reference)	1 (reference)
30 - 39	54/114	(32/68)	1.15 (0.75 – 1.74)	1.19 (0.78 – 1.83)
40 - 49	41/76	(35/65)	1.32 (0.83 – 2.10)	1.35 (0.84 – 2.16)
50 - 59	37/46	(45/55)	**1.95 (1.17 – 3.25)**	**1.85 (1.10 – 3.10)**
≥ 60	19/37	(34/66)	1.25 (0.68 – 2.32)	1.27 (0.68 – 2.38)
**Cycling frequency(trips per year)**^†^	164 vs. 145		**1.17 (1.05 - 1.36)**	**1.17 (1.05 - 1.30)**

Significant associations in bold.

* Row percent.

^†^ = continuous variable: mean trips per year for yes vs. no; odds ratio and 95% confidence interval for 52 trips per year (equivalent to cycling once per week).

An array of variables were associated with helmet use (Table 5). Participants in Toronto, where there is no legal requirement for adults to wear helmets, were less likely to wear them (59%) than those in Vancouver (76%). Trip characteristics positively associated with helmet use included use of a bike light, longer trip distances, and use of a hybrid style of bicycle. Cruiser bike use and consumption of alcohol in the 6 hours prior to the trip were associated with lower odds of helmet use. Personal characteristics positively associated with helmet use included female sex, older age, higher income and higher education.

**Table 5:** Associations between whether helmet was worn and trip or personal characteristics, variables retained in adjusted analysis only, each variable on its own (unadjusted) and in multiple logistic regression (adjusted)

	**Helmet worn**			
	**Yes/No**	(% Yes/% No)*	**Unadjusted Odds Ratio (95% Confidence Interval)**	**Adjusted Odds Ratio (95% Confidence Interval)**
**City**				
Vancouver	315/99	(76/24)	1 (reference)	1 (reference)
Toronto	163/113	(59/41)	**0.46 (0.33 – 0.64)**	**0.38 (0.25 – 0.57)**
**Bike light turned on during trip**				
No	376/179	(68/32)	1 (reference)	1 (reference)
Yes	102/33	(76/24)	1.45 (0.93 – 2.25)	**2.02 (1.17 – 3.50)**
**Trip distance**				
< 2 km	147/102	(59/41)	1 (reference)	1 (reference)
2 - < 5 km	158/63	(71/29)	**1.63 (1.10 – 2.41)**	**1.67 (1.05 – 2.65)**
5 - < 10 km	106/32	(77/23)	**2.14 (1.34 – 3.44)**	1.67 (0.96 – 2.89)
10 - < 20 km	36/12	(75/25)	1.91 (0.94 – 3.85)	1.47 (0.65 – 3.34)
≥ 20 km	31/3	(91/9)	**6.75 (2.01 – 22.7)**	**5.43 (1.42 – 20.8)**
**Bike type used on trip**				
Mountain bike	139/67	(67/23)	1 (reference)	1 (reference)
City bike	17/12	(59/41)	0.66 (0.30 – 1.46)	0.82 (0.33 – 2.05)
Touring/road bike	98/46	(68/32)	1.01 (0.64 – 1.61)	1.11 (0.65 – 1.90)
Racing bike	50/16	(76/24)	1.45 (0.77 – 2.73)	1.22 (0.58 – 2.58)
Folding bike	7/5	(58/42)	0.65 (0.19 – 2.12)	0.84 (0.20 – 3.44)
Hybrid	152/28	(84/16)	**2.58 (1.55 – 4.27)**	**2.08 (1.18 – 3.68)**
Cruiser	6/17	(26/74)	**0.17 (0.07 – 0.47)**	**0.15 (0.05 – 0.46)**
BMX bike	1/6	(14/86)	**0.07 (0.01 – 0.66)**	0.15 (0.02 – 1.40)
Fixed gear	8/15	(35/65)	**0.25 (0.10 – 0.61)**	0.42 (0.16 – 1.16)
**Alcohol used in 6 hours prior to trip**				
No	442/175	(72/28)	1 (reference)	1 (reference)
Yes	36/37	(49/51)	**0.39 (0.24 – 0.64)**	**0.42 (0.23 – 0.78)**
**Sex**				
Male	272/138	(66/34)	1 (reference)	1 (reference)
Female	206/74	(74/26)	1.33 (0.95 – 1.87)	**1.62 (1.06 – 2.48)**
**Age**				
19 - 29	160/103	(61/39)	1 (reference)	1 (reference)
30 - 39	114/54	(68/32)	1.33 (0.88 – 2.01)	0.98 (0.59 – 1.63)
40 - 49	90/27	(77/23)	**2.14 (1.29 – 3.53)**	1.27 (0.67 – 2.40)
50 - 59	69/14	(83/17)	**3.04 (1.62 – 5.68)**	**2.45 (1.12 – 5.35)**
≥ 60	44/12	(79/21)	**2.24 (1.27 – 4.45)**	1.35 (0.58 – 3.17)
**Income**				
< $15,000	30/36	(45/55)	**0.44 (0.24 – 0.81)**	**0.45 (0.22 – 0.92)**
$15,000 - 29,999	50/33	(60/40)	0.75 (0.42 – 1.33)	0.94 (0.48 – 1.83)
$30,000 - 49,999	83/37	(69/31)	1.13 (0.66 – 1.92)	1.12 (0.61 – 2.07)
$50,000 - 79,999	88/45	(69/31)	1 (reference)	1 (reference)
$80,000 - 119,999	80/19	(81/19)	**2.11 (1.14 – 3.90)**	1.67 (0.84 – 3.35)
≥ $120,000	97/12	(89/11)	**4.00 (1.99 – 8.06)**	**2.28 (1.03 – 5.07)**
DK/Refuse	50/30	(63/37)	0.89 (0.50 – 1.61)	0.84 (0.42 – 1.71)
**Education**				
Some high school	7/6	(54/46)	0.43 (0.13 – 1.42)	0.42 (0.11 – 1.70)
Completed high school	19/21	(48/52)	**0.30 (0.15 – 0.59)**	**0.30 (0.13 – 0.68)**
Some post-secondary education	71/48	(60/40)	**0.47 (0.29 – 0.76)**	0.61 (0.35 – 1.06)
Completed college/ technical diploma	68/57	(54/46)	**0.38 (0.24 – 0.61)**	**0.43 (0.25 – 0.74)**
Completed university degree	190/59	(76/24)	1 (reference)	1 (reference)
Completed graduate degree	123/21	(85/15)	**1.80 (1.04 – 3.12)**	1.29 (0.70 – 2.40)

Significant associations in bold.

* Row percent.

## Discussion

The most commonly used safety equipment was helmets (69% overall), even in Toronto where use of helmets is not required of adults. This reflects the emphasis on helmets as “the major safety measure for bicyclists” in Canada [27]. Use of lights was uncommon (~20%), but it is required at night in both jurisdictions, and these laws were followed by about the same proportion of cyclists as complied with helmet legislation in Vancouver (~77%). Use of lights at dusk and dawn is also mandated by legislation, but this was much less prevalent in our study. The use of lights in the daytime was rare. Since 1990, the Canadian Motor Vehicle Safety Standard has required that all motor vehicles be equipped with front daytime running lights, so it is interesting that the potential for increasing the visibility of cyclists via use of lights in daytime has not been recognized either in law or by individuals. A new development related to this issue is bike share systems. These are being implemented in various Canadian cities and, to date, all have bikes equipped with front and rear LED lights that are on whenever the bicycle is moving. Bike share systems were not in place in Toronto or Vancouver at the time of our study. Use of conspicuous colours (white, yellow, orange or red) on the torso (33%) was more common than use of lights in daytime, however the majority of participants wore other colours. Poor weather was associated with less use of conspicuous clothing, opposite to what would be desirable, perhaps indicative of the typical colours of coats sold for cold or rainy weather. It is possible that some of the dark or muted coloured coats had reflective tape that would be visible when illuminated at night, but we did not document this in a systematic way. Brightly coloured jackets are sold in bicycle shops, and these may be more often purchased by frequent cyclists; they were more likely to wear conspicuous clothing in this study.

Of the three types of safety equipment examined here, helmets have been the most frequently studied. Studies that elicited self-reported *regular* use of helmets in Oregon and New Zealand indicated very high proportions (95% or more) [19,21], but these levels seem unrealistically high compared to observations of cyclists in the field and self-reports about a specific trip (e.g., an injury trip, as in this study). In US and Canadian jurisdictions, typical proportions of adults wearing helmets have been in the range of 30 to 50% where there is no legal requirement to do so [10-12,15,16,18,22,23,25], and somewhat over 70% where legislation requires use by adults [16]. These proportions are comparable to (though slightly lower than) our findings, perhaps because our sample was skewed to regular cyclists. In continental Europe, helmet use rates are considerably lower, with reports of 2% in Paris [15], 12% in Germany [14], and 2-6% among pediatricians in the Netherlands [24]. A UK study reported 27% of observed cyclists wore helmets [20]. Factors associated with *not* wearing a helmet are similar to many of those found in our study: alcohol use [10-12]; younger ages [14,16,25]; lower education and income [14,16]; and less distance or duration of cycling [14,15,25]. Studies examining sex have not found consistent relationships [14-16,22], though in North America (as in our study) women appear to be more likely to use helmets [15,16].

Studies of the prevalence of light use have mainly focused on use at dawn, dusk and night, rather than during the day. Several have surveyed self-reported regular use and may suffer from over-reporting: 92% indicated back light and 87% front light use in New Zealand [21]; 90% back light and 83% front light use in Australia [13]; 96% any light use in Portland, Oregon [19]. Those doing direct field observations have found lower proportions: 50% rear light use, 48% front light use in the UK [20]; 40 to 60% use at night in New Zealand [17]. One study compared Paris and Boston and found that 47% of cyclists used lights at night in Paris *versus* 15% in Boston [15]. Factors associated with light use were rarely studied, but included results similar to ours: light use was more common among those who wore helmets [20]; and among men, older adults, and on weekdays [15].

Few studies have examined conspicuous clothing use and those that have suggest it is less common than use of helmets or lights at night. The clothing colours and types studied were not always defined or similar. A study in Alberta, Canada observed 16% of cyclists wearing yellow, orange or red clothing on the torso, and 19% wearing white [18]. A study in the UK observed 10% wearing fluorescent or reflective clothing [20]. In self-report studies, 30% reported regular use of fluorescent colours in New Zealand [21] and 23% reported always use in Australia [13]. Only one study examined features associated with use and they found highly visible clothing to be associated with helmet use [20]. In our study, conspicuous clothing use and helmet use were not associated.

As with studies of equipment use, studies of injury prevention related to safety equipment have focused on helmets. Enough studies have examined the association between helmets and injuries to allow reviews and meta-analyses. Helmets have been shown to reduce head and face injuries (and increase neck injuries) in the event of a crash [28] and this type of post-crash protection has been emphasized in North America. In contrast, there has been little research directly examining the effectiveness of lights or conspicuous clothing as a means of preventing crashes. This may be because this type of study is much more difficult to conduct than studies of injury type and severity that dominate the literature on helmets. A New Zealand study [21] found that cyclists who reported always wearing fluorescent colours had lower risks of crashes and days off work. They also found a lower crash risk among those who reported always using a back light at night. Kwan and Mapstone [29] reviewed the literature on visibility aids for cyclists and pedestrians. They concluded that daytime visibility improved with white, yellow, orange, and red materials, and that night-time visibility aids (lights especially, but also reflective clothing) enhanced detection and recognition and shortened reaction times of observers. More recent studies support these results [13,18]. Our study design did not allow analyses of the risk of crashes with the various types of safety equipment reported here. We were able to examine various surrogates of severity (e.g., transport by ambulance, hospitalization) and found that none of these safety equipment types was associated with injury severity, after controlling for factors such as route infrastructure, weather, and demographics [30].

This study had a number of limitations. It collected data from injured cyclists who attended a hospital emergency department, so may not be representative of all cyclists. The cyclists in this study, despite being recruited via an injury study and despite having many potential participants who were not contactable, mirror characteristics of cyclists in North America, i.e., dominantly male, young, and educated [3,31]. An exception is that the sample included mainly frequent cyclists, likely a reflection of the fact that more time spent cycling offers greater opportunity to be injured. The study used self-reports by injured cyclists about safety equipment use. Our results compared favourably to studies using observations of cyclists in the field [10–12,15- 18,20,22,23,25]. This may be because self-reporting about a single trip (in this case, the injury trip) is well recalled and accurately reported. In addition, we used deliberate question ordering and wording to reduce the chance that responses aimed at conforming to behavioural norms and laws. For example, we purposely asked about helmet use indirectly by querying the colour of the helmet, instead of whether a helmet was worn. Other questions that might be affected by social desirability bias are those on alcohol and drug use. In our study, 10% of cyclists self-reported drinking in the 6-hour time frame prior to the trip. In studies that measured blood alcohol levels in more severely injured adult cyclists (fatally injured or hospitalized), 10, 14 and 19% had levels over 0.08 g/dL [11,23,32], suggesting that our mode of questioning may have produced reasonable results for alcohol use as well. We did not collect data on the reasons why safety equipment was used or not, but we were able to examine an extensive list of personal and trip characteristics associated with equipment use. Finally, we did not collect information about many other types of individual-based equipment that may prevent injuries to cyclists, including reflective tape, reflectors, rear-view mirrors, disc *versus* rim brakes, and bells.

## Conclusions

In this study of injured cyclists in two of Canada’s largest cities, we examined three types of individual-based safety equipment and found that helmets were the most frequently used, and were the only type of equipment used on the majority of trips. Helmets are a post-crash injury mitigation measure, whereas visibility aids are meant to allow other route users to detect and avoid a cyclist and thus prevent crashes from occurring. Studies of the injury reduction effectiveness of these pre-crash primary prevention devices are promising but rare, so this is an area worthy of further study. In the meantime, there is room for increasing awareness among cyclists and cycling stakeholders of the enhanced detection provided by visibility aids and their potential to reduce collision risk.

There were groups in the cycling population who were less likely to use each type of safety equipment, suggesting areas of focus for change. People who tended not to use bike lights or conspicuous clothing were those who cycle less and may have less knowledge about cycling equipment (e.g., weekend cyclists, less frequent cyclists). This suggests the potential value of communication campaigns like the ones that have increased helmet use. Another approach could include changes to bicycle sales, so that all commuter bikes are sold with lights (as motor vehicles are). The population not wearing helmets is smaller and may be more difficult to reach with additional messaging: those associated with risk-taking behaviour (youth, men, people who have consumed alcohol); and those with less income and education. Strategies to prevent injuries in such populations may require a different focus: safety improvements in the cycling environment (e.g., lower motor vehicle speed limits on residential streets, dedicated bicycle infrastructure including cycle tracks, bike lanes and paths) [8,26,33]. Such population-level approaches to injury prevention benefit all cyclists and may benefit other road users as well.

## Competing interests

CCOR, MW, and PAC have held consultancies to related to their transportation or injury biomechanics expertise. PAC has stock in a company developing a helmet that he co-invented. All other authors have no financial or other relationships or activities that could appear to have influenced the submitted work.

## Authors’ contributions

KT, MAH, CCOR, and PC were responsible for initial conception and design of the study. KT, MAH, CCOR, PC, MW, MC, MDC, JB, GH, SB and SMF were responsible for the funding proposal. MAH, CCOR, MW, MM, MDC and KT designed and tested data collection instruments. JB, GH, SMF, and MDC contributed to identification of injured cyclists at the study hospitals. HS was responsible for data analyses. KT drafted the article. All authors contributed to study design and implementation, analysis decisions, interpretation of results, and critical revision of the article.

## Pre-publication history

The pre-publication history for this paper can be accessed here:

http://www.biomedcentral.com/1471-2458/12/765/prepub
